# Climate Change Adaptation to Smoke Haze for Improved Child Health in Southeast Asia: Analysis Situation in Palembang, Indonesia

**DOI:** 10.5334/aogh.5211

**Published:** 2026-06-08

**Authors:** Budi Haryanto, Fitri Kurniasari, Indang Trihandini, Fajar Nugraha, Nabila Gayatri Widayana, Nurul Laksmi Winarni

**Affiliations:** 1 Department of Environmental Health, Faculty of Public Health – Universitas Indonesia; 2 Research Center for Climate Change, RCCC – Universitas Indonesia, Depok, Indonesia; 3 Department of Biostatistics and Population, Faculty of Public Health – Universitas Indonesia

**Keywords:** haze, forest and peatland fires, children’s health, respiratory diseases, PM_2.5_, Palembang

## Abstract

*Background:* Haze from forest and peatland fires is a major recurring hazard in Indonesia. During the 2015 fire crisis, more than 43 million people were affected, with PM_10_ concentrations reaching 2,108.5 μg/m³ in Palangkaraya, while children were among the most vulnerable populations due to their increased susceptibility to air pollution.

*Objective:* This study assessed the impact of forest and peatland fire haze on child respiratory health in Palembang, Indonesia, by examining associations between ambient concentrations of PM_10_, PM_2.5_, and SO_2_ and the incidence of acute respiratory infection (ARI) and pneumonia to inform child-focused air quality management and public health preparedness.

*Methods:* An ecological time-series study was conducted using data from 2011 to 2020 in Palembang, a city consistently affected by annual peatland fires. The dataset included 366,632 ARI cases and 27,574 pneumonia cases among children. Ambient air quality data were obtained from the Indonesian Meteorology, Climatology and Geophysics Agency (BMKG). Pearson correlation analyses with a one-month lag were performed, followed by linear regression modeling.

*Results:* Particulate matter showed strong associations with increased childhood respiratory morbidity. Pneumonia demonstrated more consistent relationships with PM_2.5_, reflecting the deeper penetration of fine particles into the respiratory tract. Regression models indicated that rises in PM_2.5_ predicted higher pneumonia incidence, while PM_10_ was more consistently associated with ARI. Associations involving SO_2_ were less consistent, though it may act synergistically with particulate matter to worsen respiratory outcomes.

*Conclusion:* Haze exposure significantly elevates the risk of respiratory illness in children. PM_2.5_ is strongly linked to pneumonia, whereas PM_10_ is more closely related to ARI. The persistent nature of haze and children’s heightened vulnerability highlight the need to integrate child-centered risk mitigation into national air quality management and disaster preparedness strategies.

## Background

Haze resulting from forest and peatland fires remains one of the most significant environmental and public health hazards in Indonesia and Southeast Asia. Although large-scale fire events have occurred intermittently for decades, recent syntheses confirm that Indonesia continues to be among the countries most affected by landscape fires, particularly those involving peatlands, which produce dense and persistent smoke [1, 2]. Fire-related haze episodes are increasingly recognized not only as acute disasters but also as recurrent public health threats linked to land-use change, climate variability, and prolonged dry seasons.

The 2015 forest and peatland fires represent a landmark episode illustrating the scale and severity of haze exposure in Indonesia. Subsequent assessments by international agencies estimate that more than 40 million people across Indonesia were exposed to unhealthy air quality during this event, as fires burned over 2 million hectares of land, predominantly in Sumatra and Kalimantan [1, 3]. Air quality monitoring during the peak of the fires documented extreme particulate matter concentrations in several cities, including Palembang, Pontianak, and Palangkaraya, with PM_10_ levels far exceeding national and international air quality guidelines [4]. These conditions prompted emergency declarations in multiple provinces and highlighted the limited capacity of health and disaster management systems to respond to prolonged haze exposure [5].

The impacts of Indonesian haze events extend beyond national borders. Regional assessments by ASEAN and the United Nations Environment Programme confirm that transboundary haze continues to affect neighboring countries, such as Malaysia, Singapore, and Thailand, driven by long-range transport of fine particles under prevailing wind conditions [6, 7]. This underscores the regional nature of the problem and the need for coordinated mitigation and response strategies.

Smoke from forest and peatland fires is a complex mixture of pollutants, including carbon dioxide (CO_2_), carbon monoxide (CO), nitrogen oxides (NOx), volatile organic compounds (such as formaldehyde and acrolein), water vapor, and large quantities of suspended particulate matter. The chemical composition of haze varies depending on fuel type, combustion temperature, moisture content, and meteorological conditions, with peat fires producing especially high emissions of fine particles and toxic compounds [3, 8]. Recent reviews emphasize that particulate matter, particularly PM_2.5_, is the most critical component from a health perspective due to its ability to penetrate deep into the respiratory tract and enter the bloodstream.

Exposure to haze is not limited to populations living near fire sources. Fine particles can remain suspended in the atmosphere for extended periods and travel hundreds of kilometers, resulting in widespread exposure across urban and rural areas [2, 3]. PM_10_ and PM_2.5_ are commonly used indicators of haze exposure, with forest fire smoke dominated by PM_2.5_, which is strongly associated with adverse respiratory and cardiovascular outcomes.

A substantial body of epidemiological evidence links exposure to fire-related particulate matter with eye and airway irritation, reduced lung function, exacerbation of asthma, increased hospital admissions, and elevated cardiovascular risk. Symptoms commonly reported during haze episodes include coughing, wheezing, chest tightness, shortness of breath, dizziness, and fatigue, with severity influenced by exposure duration, pollutant concentration, and individual vulnerability [9, 10].

Children represent a particularly vulnerable population. Ongoing lung development, immature immune and detoxification systems, higher respiratory rates, and behavioral factors increase children’s susceptibility to air pollution. Recent studies in Southeast Asia confirm stronger associations between wildfire-related particulate matter exposure and acute respiratory infections (ARI), pneumonia, and impaired lung growth among children compared with adults [11, 12].

Against this background, the present study examines the health impacts of forest and peatland fire haze on children in Palembang, one of the cities most consistently affected by annual peatland fires in Indonesia. Specifically, the study analyzes the association between ambient concentrations of PM_10_, PM_2.5_, and sulfur dioxide (SO_2_) and the incidence of ARI and pneumonia among children, contributing to evidence needed to inform air quality management, public health preparedness, and child-focused protection strategies.

## Method

This study employed an **ecological design** to examine the association between haze-related air pollutants (PM_10_, PM_2.5_, and SO_2_) and childhood respiratory diseases. The focus was on ARI and pneumonia.

The research concentrated on the highly affected city in Indonesia, **Palembang (South Sumatra)**. This specific location was selected because it experienced **frequent and severe haze exposure** due to its proximity to forest and peatland fire hot spots. This approach allowed the study to capture city-wide exposure trends and identify associations at the population level over a long period (2011–2020). In terms of scale, the study involved a total of **366,632 cases of ARI** and **27,574 cases of pneumonia in children**.

### Data sources

This study drew on two principal data sources. First, ambient air quality data were obtained from the Indonesian Meteorology, Climatology and Geophysics Agency (BMKG), which routinely monitors concentrations of particulate matter with an aerodynamic diameter less than 10 µm (PM_10_) and less than 2.5 µm (PM_2.5_). These indicators were selected as they constitute the predominant and most health-relevant components of haze. Daily measurements were aggregated into monthly averages to ensure comparability with available health records.

Second, health outcome data were sourced from District Health Offices and community health centers (Puskesmas) in Palembang. Between 2015 and 2020, records documented 33,517 cases of pneumonia and 804,467 cases of ARI among children aged 1–12 years in Palembang. These diagnoses, derived from outpatient visits, were selected because they represent the most prevalent and sensitive respiratory outcomes associated with haze exposure.

## Data Analysis

The analysis proceeded in two stages. First, Pearson correlation coefficients (r) were calculated between monthly pollutant concentrations (PM_10_, PM_2.5_, and SO_2_) and respiratory disease cases, applying a one-month lag to account for possible delayed health effects of exposure. Correlation values were interpreted based on strength and direction, with statistical significance determined at *p* < 0.05 and *p* < 0.01. Prior to correlation analysis, data distributions were examined for normality and outliers, and scatter-plots were inspected to confirm linear relationships between pollutant concentrations and respiratory outcomes.

Second, linear regression models were constructed to further assess the predictive relationship between pollutant concentrations and disease outcomes. Separate models were developed for each city and disease type, with pollutant levels as independent variables and case counts as dependent variables. Regression diagnostics were performed to assess model validity. Residuals were inspected for normality using Q–Q plots and the Shapiro–Wilk test, while homoscedasticity was evaluated through residual-versus-fitted plots. Independence of errors was assessed using the Durbin–Watson statistic. Multicollinearity among pollutants was examined using variance inflation factors (VIF), with values < 5 considered acceptable.

### Ethical considerations

This study was based on secondary, aggregated data from official government sources. No individual patient identifiers were used, and analyses were conducted at the population level. As such, ethical clearance requirements were minimal; however, approval and permission for data use were obtained from the relevant health authorities and academic review boards.

### Rationale for approach

The ecological design allowed the study to capture city-wide exposure trends and identify associations at the population level over a long period (2011–2020). While individual-level risk factors could not be examined, this approach provided valuable insights into the public health burden of haze exposure, particularly among children, who are recognized as a vulnerable population.

## Results

The correlation analysis between PM_10_ concentrations and ARI among children in Palembang (366,632 cases) from 2011 to 2020 revealed notable spatial and temporal variations. In **Palembang**, associations were generally weaker and more variable, with both positive and negative values across the period. The strongest positive link was seen in 2017 (r = 0.12) and 2019 (r = 0.15), while slight negative correlations emerged in 2015 and 2020. This result confirms the critical role of PM_10_ in driving childhood respiratory morbidity.

From 2014 to 2020, correlations between haze pollutants and children’s respiratory health revealed mixed but important patterns (Table 1). ARI showed weak or negative associations with PM_10_, with consistent negative correlations from 2017 to 2020. Pneumonia was more strongly linked to PM_2.5_, particularly in 2014, when a significant negative correlation indicated fine particles’ critical role in respiratory outcomes. Sulfur dioxide (SO_2_) displayed variability, with no effect in 2014–2015, but a significant positive correlation in 2018, suggesting local conditions intensified its impact. These findings highlight how particulate matter and gaseous pollutants jointly contribute to children’s vulnerability during haze episodes.

**Table 1:** Statistic correlation (r) between PM_10_ and ARI, PM_2.5_ and Pneumonia, SO_2_ and Pneumonia per year in Palembang 2014–2020 (lag 1 month).

YEAR	r (ARI-PM_10_)	r (Pn-PM_2.5_)	r (Pn-SO_2_)
2014	0.027	−0.615*	NA
2015	−0.056	0.077	NA
2017	−0.286	0.046	0.574
2018	0.123	0.136	0.602*
2019	−0.287	−0.091	0.253
2020	−0.287	−0.091	0.253

*p-value less than 0.05.

Regression analyses of Palembang from 2011 to 2020 demonstrate strong associations between air pollutants and respiratory diseases (Table 2). The ARI and pneumonia were strongly influenced by PM_10_ and PM_2.5_, indicating that particulate matter plays a critical role in disease incidence.

**Table 2:** Regression analysis of PM_10_ and ARI, PM_2.5_ and Pneumonia, SO_2_ and Pneumonia in Palembang.

CITY	DISEASE	RISK FACTOR	PERIOD	REGRESSION MODEL	CONCLUSION
Palembang	ARI	PM_10_	2014–2020	ARI = 4401.27 + 11.26*PM_10_	Strong significant
Palembang	ARI	PM_10_	2014–2020	ARI = 4436.76 + 9.93*PM_10_	Strong significant
Palembang	Pneumonia	PM_2.5_	2014–2020	Pn = 334.73 + 0.655*PM_2.5_	Strong significant
Palembang	Pneumonia	PM_2.5_	2014–2020	Pn = 337.26 + 0.582*PM_2.5_	Strong significant
Palembang	Pneumonia	SO_2_	2017–2020	Pn = 332.98 + 0.026*SO_2_	Significant

This study demonstrates the substantial health impacts of forest and peatland fire haze on children in Palembang. Using air quality and health data from 2011 to 2020, strong associations were found between particulate matter and childhood respiratory diseases, particularly ARI and pneumonia, which dominate morbidity during haze episodes.

In Palembang, regression analyses confirmed that higher PM_10_ concentrations significantly increased ARI cases, especially during the intense 2014–2016 fires. Pneumonia showed even stronger and more consistent relationships with PM_2.5_ in all three cities, reflecting the deeper respiratory penetration of fine particles. Several regression models yielded highly significant results (*p* < 0.001), indicating that increases in PM_2.5_ directly predicted higher pneumonia incidence.

The role of SO_2_ was more complex: while certain years showed significant correlations with pneumonia, the direction sometimes reversed, suggesting variability in pollutant composition or lag effects. Although not the dominant driver, SO_2_ likely acts synergistically with particulate matter to exacerbate respiratory burdens.

Children’s vulnerability stems from their developing lungs, higher respiration rates, and greater outdoor activity, making them disproportionately affected by haze exposure. The peak fire years of 2015 and 2019 coincided with sharp rises in respiratory morbidity, corresponding with extreme PM concentrations exceeding safety thresholds. Overall, these findings confirm that haze-related air pollution poses a serious and recurring public health threat.

## Discussion

This study demonstrates that recurrent haze from forest and peatland fires is a major and persistent contributor to childhood respiratory morbidity in Palembang. Using 10 years of ecological data, strong associations were observed between particulate matter exposure and increased incidence of ARI and pneumonia, reinforcing haze as a significant public health hazard for children in fire-prone regions of Indonesia. Similar findings have been reported globally, where wildfire smoke exposure is consistently associated with increased pediatric respiratory morbidity and healthcare utilization [8, 9].

A key finding is the differentiated health impact by particle size. PM_10_ was more consistently associated with ARI, while PM_2.5_ showed stronger and more stable associations with pneumonia. This distinction is biologically plausible and supported by toxicological and epidemiological evidence. Coarse particles primarily affect the upper airways, whereas fine particles penetrate deep into the alveolar region, triggering inflammation, oxidative stress, and impaired immune defense [3, 13]. Systematic reviews and meta-analyses consistently identify PM_2.5_ from wildfire smoke as the most critical determinant of severe respiratory outcomes, including pneumonia and asthma-related morbidity in children [10, 14, 15].

The strong regression coefficients linking PM_2.5_ to pneumonia in Palembang underscore the particular hazard posed by peatland fires. Peat combustion produces exceptionally high concentrations of fine particulate matter with prolonged atmospheric persistence and enhanced toxicity due to high organic carbon content. Recent exposure modeling using low-cost sensor networks and regional air quality models demonstrates that PM_2.5_ concentrations during Indonesian peat fires frequently exceed national standards and WHO air quality guidelines for extended periods [4, 16]. These findings are consistent with national disaster assessments documenting the scale and persistence of fire-related air pollution in Indonesia [1, 5].

Temporal patterns further strengthen causal interpretation. Peak haze years, particularly 2015 and 2019, coincided with marked increases in ARI and pneumonia cases among children. Comparable surges in pediatric respiratory morbidity following major wildfire events have been documented in Australia and other regions, indicating that the health impacts observed in Palembang reflect a broader global pattern rather than isolated local effects [9, 17].

The role of sulfur dioxide (SO_2_) in this study was less consistent, with significant associations observed only in certain years. This variability likely reflects differences in combustion conditions, fuel composition, and meteorological factors influencing pollutant mixtures. Although SO_2_ was not the dominant driver of respiratory outcomes, evidence suggests it may act synergistically with particulate matter to exacerbate airway inflammation and infection risk [8, 11].

Children’s disproportionate vulnerability is central to interpreting these findings. Ongoing lung development, immature immune and detoxification systems, higher ventilation rates relative to body size, and activity patterns that increase outdoor exposure collectively heighten susceptibility to haze-related pollution [11, 13]. Evidence from cohort, case-crossover, and multi-country studies demonstrates that early-life exposure to wildfire-related PM_2.5_ increases the risk of ARI, pneumonia, and impaired lung development, with potential long-term consequences for respiratory health [12, 18–20].

Although the ecological design limits individual-level inference and adjustment for confounders such as indoor air pollution, nutrition, or vaccination status, the consistency of associations over time, strong biological plausibility, and concordance with international literature support the robustness of the findings. Ecological analyses remain particularly relevant for policy, as haze exposure and its health impacts occur at the population and city scale.

Importantly, these findings highlight persistent gaps in prevention and preparedness. Despite regional commitments under the ASEAN Agreement on Transboundary Haze Pollution, large-scale fire episodes continue to recur, reflecting limitations in enforcement and cross-sectoral coordination [6]. Global assessments further indicate that climate change, land-use practices, and prolonged dry seasons are likely to intensify landscape fire risk, increasing future haze-related health burdens without coordinated mitigation [2, 7].

## Conclusion

This study provides compelling evidence that forest and peatland fire haze significantly increases childhood respiratory morbidity in Palembang. PM_2.5_ is strongly associated with pneumonia, while PM_10_ is more closely linked to ARI, reflecting distinct exposure pathways and biological mechanisms. The recurrent nature of haze, combined with children’s heightened vulnerability, constitutes an ongoing public health emergency.

Protecting children from haze exposure requires moving beyond short-term emergency responses toward integrated strategies encompassing fire prevention, air quality management, health surveillance, and child-focused interventions. Strengthening early warning systems, embedding WHO air quality guidelines into national policy, and integrating child protection into haze and disaster preparedness frameworks are essential to reducing current and future health burdens [3, 11].
